# Concentration-Dependent Effects of Polyethylene Microplastics on Cadmium and Lead Bioavailability in Soil

**DOI:** 10.3390/toxics13100901

**Published:** 2025-10-21

**Authors:** Zhenbo Wang, Sihan Liu, Peng Zhao, Guangxin Li, Ran Duan, Chang Li, Haichao Fu

**Affiliations:** 1 College of Resources and Environmental Sciences, Henan Agricultural University, Zhengzhou 450002, China; 2 Key Laboratory of Soil Pollution Control and Remediation of Henan Province, Zhengzhou 450002, China; 3 Institute of Quality and Safety for Agro-Products, Henan Academy of Agricultural Sciences, Zhengzhou 450002, China

**Keywords:** cadmium, lead, polyethylene microplastics, soil properties, bacterial community structure, availability

## Abstract

The influence of microplastics (MPs) on the availability of soil heavy metals (HMs) is a current research hotspot, but how MPs regulate HM availability via soil properties and the bacterial community remains unclear. This study investigated the effects of polyethylene (PE) MP concentrations on soil properties, bacterial communities, surface chemistry, and the speciation of cadmium (Cd) and lead (Pb) through soil incubation. Results indicated that as PE MP concentration increased, soil pH and cation exchange capacity declined, while organic carbon concentration increased. Available phosphorus and alkali–hydrolyzable nitrogen concentrations increased at 0.1% and 1% PE MPs, but decreased at 10% PE MPs. Bacterial community indices, including Simpson, ACE, and Chao1, increased at 0.1% and 1% PE MPs but decreased at 10% PE MPs. PE MPs (0.1% and 1%) reduced DTPA–Cd/Pb, promoting their transformation into stable forms and surface complexation with oxygen–containing groups. In contrast, 10% PE MPs disrupted the formation of PbO, PbCO_3_, and Cd(OH)_2_, producing the opposite effect. The random forest model revealed that soil organic carbon and available phosphorus were the primary factors influencing DTPA–Pb and DTPA–Cd, respectively. Partial least squares path modeling demonstrated that PE MPs altered the physicochemical characteristics of soil and structure of bacterial communities, ultimately impacting transformation of Cd and Pb speciation, with these changes being highly dependent on PE MP concentration.

## 1. Introduction

The advancement of contemporary industry has resulted in a substantial discharge of heavy metals (HMs) into the soil, leading to serious pollution of agricultural ecosystems [1]. HMs in cropland can be readily absorbed by crops and enter the human body through food intake, posing a significant risk to human health [2,3,4]. Among the various HMs, lead (Pb) and cadmium (Cd) are particularly concerning due to their chronic and acute toxicological effects on animals and plants [5,6]. Soil serves as a primary reservoir for various pollutants, including HMs and other contaminants. Polyethylene (PE) is the most widely used plastic polymer globally, demonstrating significant application value in greenhouse construction and plastic mulching [7]. Due to its difficulty in being fully recycled and reused, significant accumulation of these plastics has occurred in the environment. Plastic waste undergoes a range of physical, chemical, and microbiological processes that generate particles smaller than 5 mm, collectively referred to as microplastics (MPs), among which polyethylene microplastics (PE MPs) are a major type [8,9]. MPs are widely distributed across various land use types globally, with abundance ranges of 0–10^6^ items/kg and 0–10^5^ mg/kg. Agricultural soils exhibit a higher prevalence of MP contamination (61%) [10]. Liu et al. [11] reported that the abundance of MPs in agricultural soils has reached levels ranging from 4350 items/kg to 24,120 items/kg. Notably, low–density polyethylene (LDPE) plastic, with a size of 60 μm, requires 300 years to fully degrade in soil [7], indicating that MPs in soil gradually accumulate, leading to long–term pollution. Furthermore, the rapid increase in plastic production and mineral resource exploitation has resulted in the spatial overlap of MP and HM pollution [12]. Therefore, investigating the relationship between PE MPs and the availability of soil HMs is of paramount importance.

Soil physicochemical properties, including pH, cation exchange capacity (CEC), and soil organic carbon (SOC), are key regulators of heavy metal behavior in soils. pH influences the adsorption sites, coordination characteristics, chemical structure, and surface stability of HMs in soil [13], thereby altering their morphological properties and availability [14]. Furthermore, a decrease in CEC leads to increased availability of HMs in the soil [13]. SOC, rich in ligands and functional groups, plays an essential role in forming complexes with metal ions, which in turn influences the transport and transformation of HM forms in the soil environment [15,16]. Previous research has demonstrated that MPs alter the effectiveness of HMs in soil ecosystems, with the degree and direction of these effects contingent upon the concentration levels of MPs. For instance, Wang et al. [17] demonstrated that 10% polyethylene supplementation significantly decreased DTPA–extractable Cd in soil compared to 0.1% and 1% PE MPs supplementation. Conversely, another study indicated that 10% PE MPs resulted in elevated available Cd levels in soil compared to lower doses [14]. The findings from these studies are inconsistent; thus, the joint impacts of MPs and HMs require further examination, particularly concerning the possible concentration–effect correlation.

The presence of HMs and MPs in soil can exert various direct or indirect effects on soil microorganisms [18]. Research indicated that simultaneous exposure to plastic microfibers (PMFs) and Cd leads to an increased relative abundance of the bacterial genus *Sphingomonas*, while concurrently suppressing iron–oxidizing bacteria [19]. A study conducted by Tang et al. [20], polyethylene terephthalate (PET) MPs decrease the accessibility of HMs but also change the makeup of soil microbial communities in soils contaminated with HMs. Furthermore, findings by Jiang et al. [21] revealed that the presence of both MPs and Cd in the soil environment disrupts the structure of bacterial communities. Additional evidence from Feng et al. [22] suggested that traditional MPs and biodegradable MPs not only restructure microbial communities in soils contaminated with HMs but also interfere with functional genes associated with exogenous biodegradation and metabolism, consequently affecting the availability of lead (Pb) and zinc (Zn). Previous research has primarily focused on the external addition of HMs to soil [19,22,23], but how MPs affect bacterial communities in situ Cd–Pb contaminated soils has rarely been investigated.

Previous research has produced contradictory conclusions on how MPs affect the bioavailability of HMs in soils, with some studies reporting decreases [17], others increases [14], and some no significant effects [19]. Moreover, most existing work has overlooked the potential concentration–dependent effects of MPs and rarely integrated soil physicochemical properties, microbial communities, and molecular–level mechanisms into a single framework. To address this gap, the present study (i) reveals a concentration–dependent regulation of Cd and Pb bioavailability by polyethylene MPs, showing opposite effects at low versus high doses of PE MPs; (ii) employs a combination of soil chemical indices, bacterial community analysis, and advanced spectroscopic techniques (Fourier transform infrared spectroscopy, X–ray photoelectron spectroscopy, X–ray diffraction) to elucidate the underlying mechanisms; and (iii) applies random forest and partial least squares path modeling to disentangle the relative contributions of soil properties and microbial communities to HM availability. This integrative approach fills an important research niche by clarifying how varying concentrations of polyethylene MPs regulate HM speciation and availability in co–contaminated agricultural soils.

## 2. Material and Methods

### 2.1. PE MPs and Soils

The PE MPs were characterized before the incubation experiment (Figure S1). Their morphology was examined using scanning electron microscopy (SEM, ZEISS Sigma 300, Oberkochen, Germany) and confirmed to consist of irregular fragments measuring between 5 and 15 µm (Figure S1A). Fourier transform infrared spectroscopy (FTIR, Nicolet iS10, Thermo Fisher Scientific, Waltham, MA, USA) was employed to verify the polymer composition by matching the characteristic absorption bands of polyethylene at approximately 2915, 2864, 1470, and 717 cm^−1^ (Figure S1B) [24].

The Cd– and Pb–contaminated soil used in this study was collected from agricultural land in Jiyuan City, Henan Province, China. The samples were taken from the topsoil (0–20 cm), and the tested soil type was cinnamon soil. The concentrations of available Cd and Pb in the soil were 1.22 mg/kg and 56.39 mg/kg, respectively. The soil was allowed to dry in the air at room temperature before being sifted through a 2 mm mesh for further use. Table S1 displays the physicochemical properties of the soil sample.

### 2.2. Soil Incubation Experiment

Soil incubation experiments were conducted with four treatments: 0% PE MPs (control, CK), 0.1% PE MPs (0.1%), 1% PE MPs (1%), and 10% PE MPs (10%), each with twelve replicates, requiring 200 g of dry soil per repetition. Specifically, MPs were thoroughly ground and mixed with a small quantity of soil, which was then gradually incorporated into the remaining 180 g of soil. This mixture was stirred repeatedly with a glass rod for 20 min to ensure even distribution of the MPs throughout the soil. During the incubation period, deionized water was added regularly to maintain 60% of the optimal water–holding capacity. Soil samples were collected after 10, 30, 60, and 90 days, with three replicates randomly collected from each sampling time. At 90 days, fresh soil samples were obtained for bacterial diversity analysis, while the leftover samples were air–dried, sifted through 20–mesh and 100–mesh sieves, and subsequently stored in bags for later use.

### 2.3. Soil Physicochemical Analysis

Soil pH was measured using a pH meter with a suspension ratio of 1:2.5 (soil: water, *w*/*v*) [25]. The diffusion method was employed to determine alkali–hydrolyzable nitrogen (AH–N) [26]. To extract soil available phosphorus (AP), sodium bicarbonate was utilized, and its concentration was then measured using the molybdenum–antimony colorimetric technique [26]. For evaluating the soil cation exchange capacity, the spectrophotometric method involving hexamminecobalt trichloride solution was applied (HJ 889−2017). The quantification of SOC was conducted through the potassium dichromate–sulfuric acid oxidation method. The available Cd and Pb were obtained using a diethylene triamine penta–acetic acid (DTPA) solution at a concentration of 0.005 mol/L, with a pH of 7.3 [25], and were measured with atomic absorption spectrophotometer (ZEEnit700, Analytik Jena AG, Jena, Germany).

The approach developed by Tessier et al. [27] was employed to investigate the various forms of Cd and Pb found in the soil. Additional information regarding the analysis of Cd speciation in soil is available in Supplementary Materials Text S1.

### 2.4. Soil DNA Extraction and 16S rRNA Illumina Sequencing

Genomic DNA extraction was performed on microbial sources by utilizing soil samples processed with the E.Z.N.A^®^ Soil DNA Kit, providing the template for amplifying the hypervariable V3–V4 region of the bacterial 16S rRNA gene with primer pairs 338F and 806R. Once the amplification was complete, the PCR product was retrieved from a 2% agarose gel and purified, after which its concentration was measured using a Qubit 4.0 (Thermo Fisher Scientific, Waltham, MA, USA). The purified amplicons were combined in equal molar concentrations and subjected to paired–end sequencing on the Illumina Nextseq2000 platform (Illumina, San Diego, CA, USA), following the standard protocols established by Majorbio Bio–Pharm Technology Co. Ltd. (Shanghai, China). To maintain sample integrity, quality control of the paired–end raw sequencing reads was conducted using the fastp software (https://github.com/OpenGene/fastp (accessed on 20 July 2024, version 0.19.6). Subsequently, the processed sequences were clustered into operational taxonomic units (OTUs) with UPARSE 7.1 (http://drive5.com/uparse/ (accessed on 20 July 2024)) at a 97% sequence similarity threshold, with the most prevalent sequence in each OTU chosen as the representative sequence.

### 2.5. Characterization of Soil Components

The crystallographic structures of the soil were examined following a 90–day incubation period using X–ray diffraction (XRD, D8 Advance, Bruker, Ettlingen, Germany), which conducted at a scanning speed of 2°/min within the range of 10° ≤ 2θ ≤ 80°. Additionally, the crystallographic characteristics of the soil particles were evaluated through X–ray photoelectron spectroscopy (XPS, Thermo Scientific K–Alpha, Waltham, MA, USA). For Fourier transform infrared spectroscopy (FTIR), soil samples were air–dried and ground using an agate mortar to pass through a 100–mesh sieve. Approximately 1 mg of the finely ground soil sample was thoroughly mixed with 100 mg of spectroscopic grade KBr, ground again, and pressed into a thin sheet under vacuum. The pellets were scanned in the range of 4000–400 cm^−1^ with a resolution of 4 cm^−1^ using a Thermo Scientific Nicolet iS10 FTIR spectrometer (FTIR, Nicolet iS10, Thermo Fisher Scientific, Waltham, MA, USA) [28]. These techniques (FTIR, XRD, and XPS) were employed to identify specific changes in functional groups, mineral phases, and surface elemental composition relevant to soil–MPs–HMs interactions, as widely reported in previous studies [23,29].

### 2.6. Statistical Analysis

A one–way analysis of variance (ANOVA) was performed with the aid of IBM SPSS Statistics 26.0 software (IBM SPSS Inc., Chicago, USA), where means were evaluated using Duncan’s multiple range tests. Line graphs and circular bar plots were generated employing Origin software 2024 (OriginLab Corp., Northampton, MA, USA). Bioinformatic analysis of soil microbiota and the Mantel test were conducted through the Majorbio Cloud platform (https://cloud.majorbio.com (accessed on 25 July 2024)). The Spearman correlation analysis was carried out utilizing Wekemo Bioincloud (https://www.bioincloud.tech(accessed on 25 July 2024)). Random forest models were created using R software (version 4.3.2; R Core Team) to identify the key microorganisms affecting changes in the soil environment. Furthermore, Partial least squares path modeling (PLS–PM) was applied to explore potential associations among microplastic concentration, soil chemical index, alpha diversity index, and the speciation and bioavailability of Cd/Pb, also with R software.

## 3. Results

### 3.1. Effect of PE MPs on Available Cd and Pb

**Figure 1:**
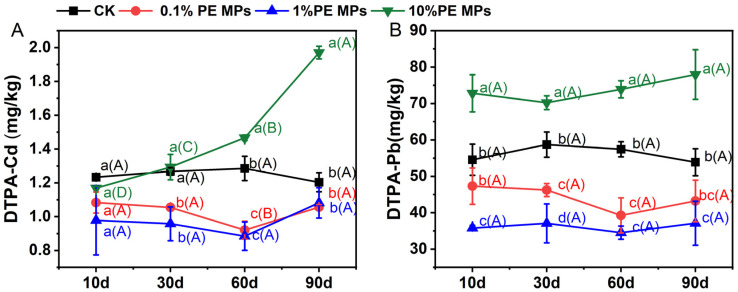
Effect of PE MPs on soil DTPA–Cd (**A**) and DTPA–Pb (**B**). Differences between various treatments at the same time points are indicated by lowercase letters, whereas uppercase letters denote differences across different time points. Data are presented as mean ± SD (*n* = 3). Error bars represent SD. The different letters denote significant differences (*p* < 0.05).

The incorporation of PE MPs has a large effect on the availability of Cd and Pb in soil (Figure 1). During the initial stages of incubation, the introduction of PE MPs resulted in lower levels of DTPA–Cd and DTPA–Pb. However, as the incubation period progressed, the application of 10% PE MPs led to a significant increase in the levels of DTPA–Cd and DTPA–Pb. Following a 90–day incubation period, compared to CK, DTPA–Cd concentrations decreased by 12.21% and 10.14% under the 0.1% and 1.0% PE MP treatments, respectively. In contrast, DTPA–Cd concentrations increased by 63.74% under the 10% PE MPs treatment (Figure 1A). The effect of PE MPs on DTPA–Pb mirrored that on DTPA–Cd. Following a 90–day incubation period, compared to CK, DTPA–Pb concentrations decreased by 19.76% and 31.10% under the 0.1% and 1.0% PE MPs treatments, respectively, while DTPA–Pb concentrations increased by 44.69% under the 10% PE MPs treatment (Figure 1B).

### 3.2. Effect of PE MPs on the Chemical Speciation of Cd and Pb

**Figure 2:**
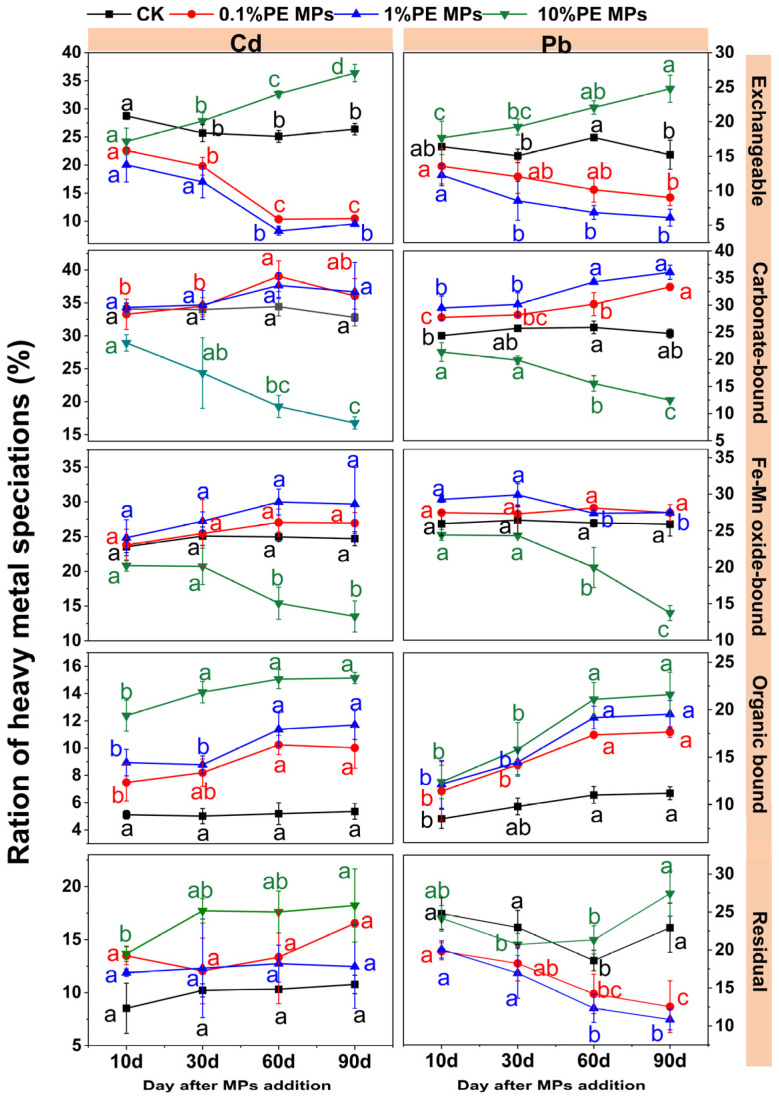
Effect of PE MPs on the chemical speciation of Cd and Pb. The data is expressed as mean ± SD (*n* = 3), with the error bars denoting the SD. The different letters denote significant differences (*p* < 0.05).

As shown in Figure 2, at 0.1% and 1% PE MPs, the exchangeable forms (F1) of Cd and Pb exhibited a gradual reduction over a 90–day incubation period. Specifically, the F1 percentage of Cd diminished from 26.35% to 10.46% and 9.51%, respectively. In contrast, the F1 percentage of Pb reduced from 15.21% to 9.02% and 6.11%, respectively, when compared to CK. At high concentration (10%), the F1 of Cd and Pb gradually increased, whereas after 90 days of incubation, the F1 of Cd and Pb increased from 26.35% to 36.35%, and from 15.21% to 24.78%, respectively, compared to CK. The carbonate–bound (F2) of Cd and Pb increased at 0.1% and 1% PE MPs but decreased at 10% PE MPs. The Fe–Mn oxide–bound (F3) of Cd and Pb decreased more significantly at 10% PE MPs. The (organic–bound) F4 of Cd and Pb was lowest in CK treatment, gradually increasing with high concentration of PE MPs and longer incubation times. After 90 days incubation phase, the F4 of Cd increased from 5.34% to 10.00%, 11.68%, and 15.13%, while the F4 of Pb increased from 11.21% to 17.65%, 19.53%, and 21.60%, respectively, compared to CK. The residual (F5) of Cd was lowest in the CK treatment and highest at 10% PE MPs, whereas the F5 of Pb was lower at 0.1%, 1% PE MPs and highest at 10% PE MPs.

### 3.3. Effects of PE MPs on Soil Physicochemical Properties

**Figure 3:**
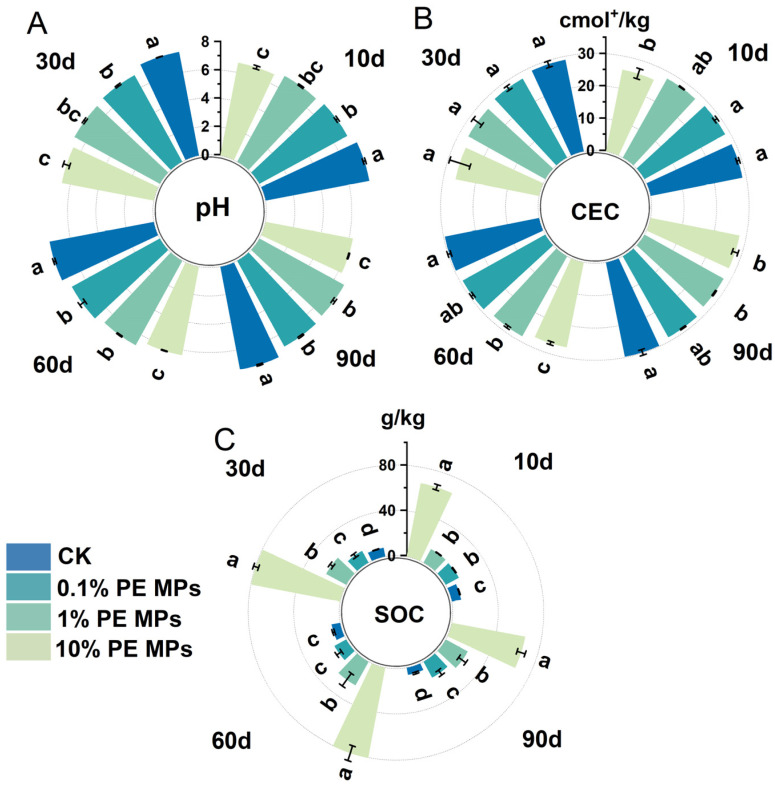
Effects of PE MPs on soil physicochemical properties ((**A**) pH, (**B**) CEC, (**C**) Soil organic carbon). The data is expressed as mean ± SD (*n* = 3), with the error bars denoting the SD. The different letters denote significant differences (*p* < 0.05).

The incorporation of PE MPs altered the characteristics of the soil. As illustrated in Figure 3A, soil pH consistently declined with increasing concentrations of PE MPs. After 90 days, the soil pH decreased by 6.62%, 8.24%, and 15.46% compared to the CK treatment, with statistical significance (*p* < 0.05). Under the 10% PE MPs treatment, soil pH steadily decreased over time. Specifically, compared to the pH at 10 days of incubation, the pH dropped by 0.42 units after 90 days. In contrast, the 0.1% and 1% PE MPs treatments caused minimal disturbance, and the soil pH remained stable throughout the incubation period (Figure S2). After 90 days, the 10% PE MPs treatment reduced CEC by 4.09% compared to CK. Furthermore, the SOC content significantly increased with rising PE MP concentrations (Figure 3C). After 90 days, SOC content increased by factors of 1.49, 2.42, and 9.20 compared to CK. Additionally, AH–N and AP content were also measured. As shown in Figure S3, AP content increased with 0.1% and 1% PE MPs, while the 10% PE MPs treatment had the opposite effect. Notably, the 1% PE MPs treatment significantly increased AH–N content compared to CK, whereas the 10% PE MPs treatment resulted in the lowest levels of AH–N.

### 3.4. Effects of PE MPs on the Diversity and Structure of Soil Bacterial Community

**Figure 4:**
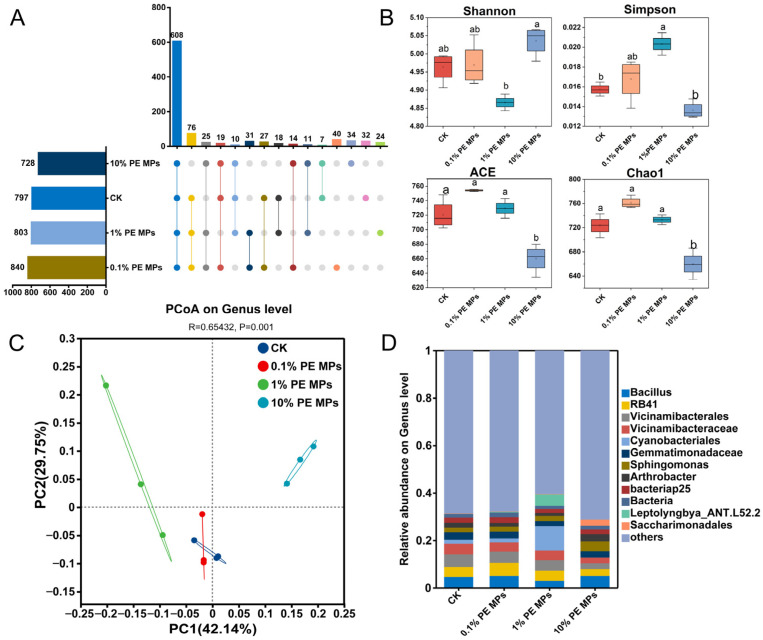
Effects of PE MPs on the diversity and structure of soil bacterial community. (**A**) Upset diagrams analysis of sediments treated with different PE MP concentrations. (**B**) Alpha diversity indices of the soil microbial community, the different letters denote significant differences (*p* < 0.05). (**C**) Principal Coordinates Analysis (PCoA) plot at the Genus level. (**D**) The relative abundance of bacterial community in soil at the genus level. Different colored nodes represent different phylum levels.

According to the results obtained from high–throughput sequencing of 16S rRNA, the quantity of OTUs at the genus level ranged from 728 in the 10% to 840 in the 0.1% PE MPs treatment (Figure 4A). The unique OTU counts for the various treatments were 32 (4.02%), 24 (2.99%), 34 (4.67%), and 40 (4.76%), with a total of 608 shared OTUs (19.19%) (Figure 4A and Figure S4). The addition of PE MPs influenced bacterial community diversity. The 1% PE MPs treatment increased the Simpson index, while the 10% PE MPs reduced both the Chao1 and ACE indices (Figure 4B). Principal coordinate analysis (PCoA) demonstrated that variations in the concentration of PE MPs, directly influenced the differences noted within the soil bacterial community (Figure 4C). At the phylum level, *Proteobacteria* and *Acidobacteriota* were the predominant phyla in the CK, 0.1%, and 1% PE MPs samples, whereas *Proteobacteria* and *Actinobacteriota* were the most prominent phyla in the 10% PE MPs sample. With 1% PE MPs, the relative abundance of *Cyanobacteria* increased, whereas under 10% PE MPs, the abundance of *Actinobacteriota* rose, and the abundance of *Acidobacteriota* declined (Figure S5B). At the genus level, bacterial communities exposed to the 0.1% PE MPs and CK treatments displayed similarities; however, the introduction of 1% PE MPs resulted in a decrease in the relative prevalence of *Bacillus* while simultaneously increasing the relative prevalence of *Cyanobacteriales* and *Leptolyngbya_ANT.L52.2*. After adding 10% PE MPs, there was an observed increase in the relative abundance of *Bacillus*, *Sphingomonas*, *Arthrobacter*, and *Saccharimonadales*, whereas the relative abundance of *RB41, Vicinamibacteraceae*, and *Vicinamibacterales* diminished (Figure 4D). According to the random forest model, *Elsterales*, *Marinococcaceae*, and *Sandaracinaceae* were identified as the three most important bacterial genera (Figure S5A). The abundance of *Brevundimonas* and *Obscuribacteraceae* increased at 0.1% PE MPs, while the abundance of *Methyloligellaceae* increased under 1% PE MPs.

### 3.5. Correlation Analysis in Soil Physicochemical Properties and Key Bacterial

**Figure 5:**
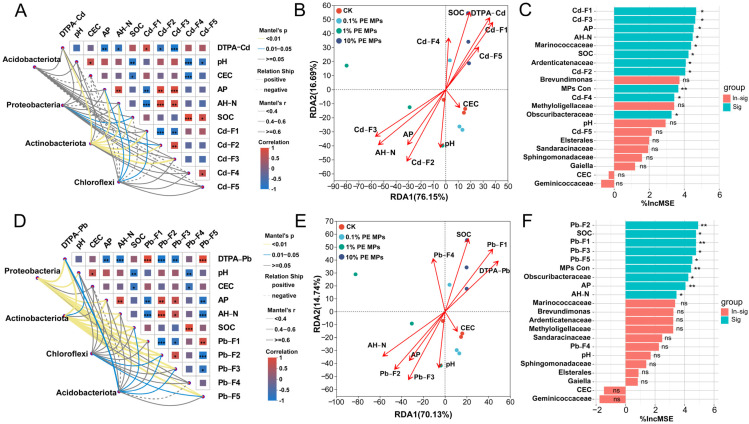
Analysis of the correlation between soil properties and bacterial community structure. Utilizing the Mantel test to examine the connection between soil physicochemical properties and the *Proteobacteria*, *Acidobacteriota*, *Actinobacteriota*, and *Chloroflexi* ((**A**) Cd, (**D**) Pb). Distribution pattern of soil physicochemical parameters by redundancy analysis (RDA) ((**B**) Cd, (**E**) Pb). Random forest analysis demonstrating the influence of soil properties and the ten most prevalent bacterial genera on DTPA–Cd (**C**) and DTPA–Pb (**F**). Significant levels are indicated: * *p* < 0.05; ** *p* < 0.01; *** *p* < 0.001.

The analysis of bacterial communities at the phylum level identified four dominant phyla: *Proteobacteria*, *Acidobacteriota*, *Actinobacteriota*, and *Chloroflexi* in the soil (Figure 5). Their correlations with environmental factors were assessed using the Mantel test. For Cd, *Actinobacteriota* significantly influenced all factors except pH, CEC, Cd–F4, and Cd–F5 (*p* < 0.01). *Chloroflexi* significantly affected AP, AH–N, and Cd–F1 (*p* < 0.05), while *Proteobacteria* significantly impacted only Cd–F2 and SOC (*p* < 0.05) (Figure 5A). In the case of Pb, neither *Proteobacteria* nor *Actinobacteriota* had a significant effect on CEC and Pb–F4 (*p* > 0.05). Conversely, *Chloroflexi* significantly influenced Pb–F2 and Pb–F5 (*p* < 0.05), and *Acidobacteriota* significantly affected DTPA–Pb, SOC, Pb–F2, and Pb–F3 (*p* < 0.05) (Figure 5D). The redundancy analysis (RDA) indicated that soil properties accounted for 92.84% of the total variance in microbial communities for Cd, with the first and second axes (RDA1 and RDA2) explaining 76.15% and 16.69%, respectively. Specifically, Cd–F1, Cd–F2, Cd–F3, SOC, AH–N, and DTPA–Cd were the primary factors influencing microbial community distribution (Figure 5B). For Pb, the total variance was explained by RDA1 and RDA2 at rates of 70.13% and 14.74%, respectively, with the primary environmental variables influencing bacterial communities being Pb–F1, Pb–F2, Pb–F3, SOC, AH–N, and DTPA–Pb (Figure 5D).

The random forest model was employed to evaluate the influence of soil physicochemical properties and top 10 bacterial genera on DTPA–Cd and DTPA–Pb. The findings demonstrated that Cd–F1, Cd–F3, AP, and AH–N were the top 4 contributors to DTPA–Cd, while the genera *Methyloligellaceae*, *Ardenticatenaceae*, and *Obscuribacteraceae* had a notable impact on DTPA–Cd (*p* < 0.05). MPs concentration was identified as an important significant factor affecting bioavailable Cd content (*p* < 0.01) (Figure 5C). For DTPA–Pb, Pb–F2, SOC, Pb–F1, and Pb–F3 were the primary factors, with the genus *Obscuribacteraceae* contributing most significantly to bioavailable Pb content (Figure 5F). Pb–F2, Pb–F1, MPs–Con (MP concentration), and AP all significantly contributed to DTPA–Pb (*p* < 0.01). The top 10 bacterial genera were also strongly correlated with soil physicochemical properties. Significant positive relationships were observed between *Marinococcaceae*, *Methyloligellaceae* and AH–N, AP, whereas negative relationships were noted with the DTPA–Cd and DTPA–Pb (*p* < 0.05). *Sandaracinaceae* showed significant negative relationships with available nitrogen and phosphorus and significant positive relationships with bioavailable Cd and Pb (*p* < 0.05) (Figure S7).

### 3.6. Characterization of Soil

**Figure 6:**
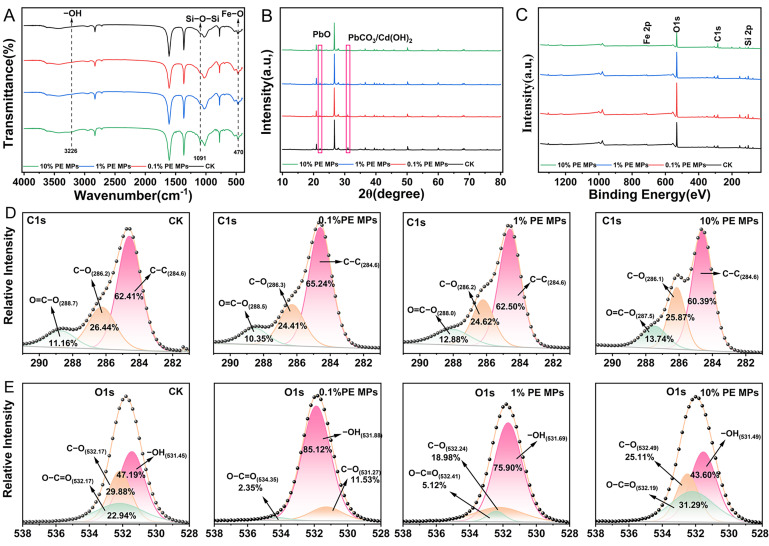
Characterization of after 90 days of incubation under different treatments. FTIR spectra (**A**), XRD patterns (**B**) and XPS spectra ((**C**) full survey; (**D**) C 1 s; (**E**) O 1 s).

**Figure 7:**
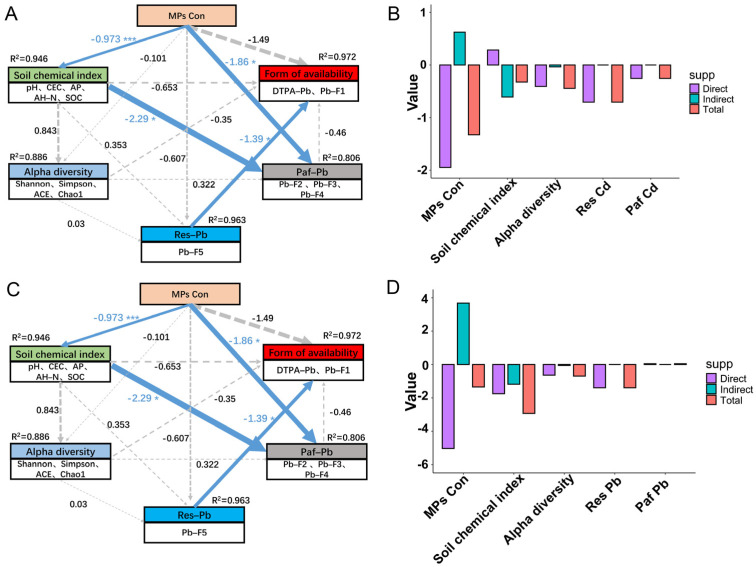
Factors regulating Cd (**A**) and Pb (**C**) availability under the influence of microplastic concentration. PLS–PM illustrates both the direct and indirect influences of observed or latent variables on the availability of Cd and Pb. Each box denotes a collection of observed or latent variables. Positive and negative causal flows are represented by blue and red arrows, respectively. Arrows with increased thickness indicate higher path coefficients. The line width reflects the size of the standardized coefficients (* *p* < 0.05, *** *p* < 0.001). The R^2^ value accompanying a parameter reflects the variance explained by other parameters. (**B**) Cd and (**D**) Pb Illustrates the distinction between direct, indirect, and total standardized effects related to the five main variables.

To further understand the effect of PE MPs in soil on the chemical form of Cd and Pb, FTIR, XPS and XRD data were collected from soil samples that were incubated for 90 days. FTIR analysis indicated an increase in the relative intensity of the –OH stretch at approximately 3226 cm^−1^ and the Si–O–Si band at around 1091 cm^−1^ under the influence of 10% PE MPs, while changes in the Fe–O band at approximately 470 cm^−1^ were also observed (Figure 6A) [29]. XRD indicated attenuation of reflections at 2θ ≈ 22.08° (PbO, PDF#76–2065) and 30.96° (PbCO_3_/Cd(OH)_2_, PDF#85–1088/PDF#71–2137) at 10% PE MPs (Figure 6B). The atomic percentages of elements in CK, 0.1% PE MPs, 1% PE MPs, and 10% PE MPs were assessed through a comprehensive XPS analysis, as presented in Table S2. The atomic percentage of C1s increases with higher PE MP concentrations, while the atomic percentages of O1s and the O/C ratios decrease accordingly. The atomic percentages of Si 2p, Al 2p, and Fe 2p exhibited a trend that initially increased before subsequently decreasing as the PE MP concentration rose. As illustrated in Figure 7C, the peak intensity of O1s, Si 2p, and Fe 2p decreased slightly under 10% PE MPs treatment, whereas the intensity of the C1s peak increased slightly. These results suggest that the presence of PE MPs may induce physicochemical interactions at the soil–PE MPs interface, primarily involving hydrogen bonding, electrostatic attraction, and surface complexation with oxygen–containing functional groups (–OH, Si–O–Si, Fe–O), thereby altering the speciation of Cd and Pb in the soil.

Through XPS fine spectrum analysis, it was observed that after incubating soil samples for 90 days, there was an increase in the peaks of O=C–O in the C1s spectra, while the peak of C–C gradually decreased under 10% PE MPs (Figure 6D). The O1s spectrum indicated that at 0.1% and 1% PE MPs, the peaks of –OH significantly increased, with the –OH content on the surface of the soil fractions rising from 47.17% to 85.12% and 78.90%, respectively. In contrast, at 10% PE MPs, the peak of –OH decreased from 47.17% to 43.60%. Conversely, at 0.1% and 1% PE MPs, the C–O and O=C–O content on the soil surface decreased from 29.88% and 22.94% (CK) to 11.53% and 18.98% (0.1% PE MPs), and 2.35% and 5.12% (1% PE MPs), respectively. However, at 10% PE MPs, there was an increase in the peaks of C–O and O=C–O in the O1s spectra compared to 0.1% and 1% PE MPs. Notably, the binding energy of the O=C–O peak decreased from 288.7 eV to 287.5 eV in the C1s spectra with increasing MP concentrations after 90 days of incubation. These findings suggest that the impacts of different concentrations of PE MPs on soil functional groups vary significantly, with the key groups that are more strongly affected being primarily C–O, O=C–O, and –OH. This variability may be a crucial factor in the alterations of soil heavy metal chemical forms influenced by different concentrations of PE MPs.

### 3.7. Analysis of Partial Least Squares Path Modeling

PLS–PM was utilized to evaluate both the direct and indirect effects of PE MP concentration, soil properties, bacterial community alpha–diversity, and the potential bioavailable form (Paf) and residual form (Res) of Cd and Pb on their availability (Figure 7). The PLS–PM analysis revealed that PE MP concentrations had a direct impact on the availability of Cd and Pb, with β coefficients of −1.33 and −1.35, respectively, and significantly influenced the physicochemical properties of the soil (*p* < 0.001). Furthermore, microbial diversity was found to significantly affect DTPA–Cd and the exchangeable forms of Cd (Cd–F1) (β = −0.48, *p* < 0.05). Additionally, the potential bioavailable Cd significantly impacted both DTPA–Cd and Cd–F1 (β = −0.254, *p* < 0.05). The concentration of PE MPs and the physicochemical properties of the soil were found to directly influence the potential bioavailable Pb (Paf–Pb), while the residue–bound Pb significantly affected the bioavailable Pb (β = −1.39, *p* < 0.05).

## 4. Discussion

### 4.1. PE MPs Altered Properties of the Soil

Soil pH is recognized as a crucial factor influencing the speciation of metals, their solubility from mineral surfaces, and their mobility and final availability [30]. This study revealed that soil pH decreases with increasing concentrations of PE MPs. Furthermore, soil pH exhibited a tendency to decline gradually over the incubation period (Figure 3A). This phenomenon may be linked to the emission of substances such as aldehydes, esters, and carboxylic acids during the degradation and aging of MPs, which could potentially influence pH levels [31,32]. Additionally, the presence of PE MPs may affect the population of ammonia–oxidizing bacteria and the nitrification process, leading to the release of hydrogen ions and a subsequent decrease in soil pH [33]. The CEC refers to the amount of cations that can be absorbed and exchanged by the soil colloids. An increase in CEC enhances ion exchange processes when soluble metal salts are adsorbed [34,35]. This study found that as the concentration of PE MPs in the soil increases, there is a corresponding gradual decline in soil CEC (Figure 3B). MPs may compete with cations for active sites on clay minerals and soil organic matter, thereby reducing CEC [36]. Furthermore, MPs may exert adsorption or desorption effects on soil organic matter, altering the structure and function of soil microbial communities. This, in turn, influences the decomposition and transformation processes of organic matter, indirectly affecting CEC [37]. Multiple mechanisms collectively determine variations in soil CEC. In the current research, the SOC content increased with rising concentrations of PE MPs during the incubation phase (Figure 3C). These results indicate that PE MPs can act as a source of SOC in the soil. Previous investigations have demonstrated that when the concentration of MPs in the soil reaches 28%, there is a significant increase in DOC levels [17,38]. Importantly, TOC and DOC present in the soil can form stable complexes with HMs. The increased SOC content in the soil further enhances the proportion of metals associated with organic matter (F4), thereby reducing the availability of HMs [39]. This conclusion is consistent with our findings (Figure 2).

### 4.2. Soil Bacterial Community Responses to Combined Exposure of PE MPs and Cd/Pb

MPs generally impact the soil microbial community through various direct and indirect mechanisms, including the release of pollutants and modifications to soil properties [40]. In this investigation, the Simpson, ACE, and Chao1 indices of the bacterial community increased under low concentrations of PE MPs (0.1% and 1%) treatments, while the addition of a high concentration (10%) of PE MPs led to a decrease in these indices (Figure 4B). The introduction of 10% PE MPs resulted in an enhanced relative abundance of *Proteobacteria*, while simultaneously causing a reduction in the relative abundance of Acidobacteriota. The abundance of *Actinobacteria* and *Chloroflexi* initially decreased and then increased with rising PE MP concentrations (Figure S6). Previous research suggests that *Proteobacteria* participate in the breakdown of PE MPs and can facilitate this process to some extent [41]. Additionally, both *Proteobacteria* and *Chloroflexi* possess genes related to HM resistance, enhancing their survival in HM–contaminated environments [42]. Recent research indicates that exposure to MPs reduces the relative abundance of the *Acidobacteriota* phylum [43]. This effect may be attributed to unstable carbon sources, given that some microbial groups in *Acidobacteriota* are more adapted to low–carbon settings. The addition of MPs may disrupt the balance of these ecosystems [44], potentially clarifying the reduction in *Acidobacteria* abundance noted in this research. A significant positive correlation has been observed between high–concentration PE MPs (7%) treatment and the relative abundance of *Actinobacteria*. This suggests that *Actinobacteria* may play a role in the degradation of MPs [45]. Previous research has demonstrated that under HM stress, HM–sensitive microorganisms tend to decline gradually, while tolerant species become more prevalent [46]. A substantial decrease in the levels of available Cd and Pb was observed at low concentrations of PE MPs (0.1% and 1%) (Figure 1), potentially mitigating the stress imposed by HMs on microorganisms. Conversely, at a 10% concentration of PE MPs, an increase in available Cd and Pb occurred, exacerbating the combined effects of PE MPs and Cd/Pb on the bacterial community. Mantel test analysis indicated a significant positive correlation (*p* < 0.01) between Actinobacteria and various forms of Cd and Pb. A previous study highlighted a favorable relationship between the presence of *Actinobacteria* in soil and the concentrations of copper (Cu), zinc (Zn), lead (Pb), and mercury (Hg) [47]. This relationship could be associated with the various HM resistance genes found in *Actinobacteria*, which assist in mitigating stress caused by HMs [48]. Random forest analysis revealed that the genera *Ardenticatenaceae*, *Marinococcaceae*, and *Obscuribacteraceae* significantly contributed to the bioavailable forms of Cd and Pb (Figure 5C,F). Pearson correlation analysis indicated that *Marinococcaceae* had a significant negative relationship with DTPA–Pb and DTPA–Cd (*p* < 0.05), while *Ardenticatenaceae* and *Obscuribacteraceae* showed significant correlations with soil pH (*p* < 0.05). These findings imply that these bacteria may have substantial implications for the behavior and distribution of Cd and Pb in the soil.

### 4.3. Effects of PE MPs on the Bioavailability and Distribution of Cd and Pb

0.1% and 1% PE MPs led to a significant decrease in both DTPA–Cd and DTPA–Pb concentrations in the soil (Figure 1). Several potential explanations for these findings are as follows: (1) MPs possess a hydrophobic carbon backbone with limited surface polarity, but environmental weathering introduces oxygen–containing functional groups (e.g., –OH, –C=O, –COOH). These groups provide negatively charged and polar sites that can bind cationic metals through electrostatic attraction and surface complexation [49]. As shown in Figure S8, PE MPs can adsorb a certain amount of Cd(II) and Pb(II), which may be attributed to the PE MPs themselves possessing a certain specific surface area and surface functional groups [50]. Additionally, the addition of PE MP reduces the adsorption of Cd(II) and Pb(II) by the soil compared to pure soil. This phenomenon may be attributed to the dilution effect of MPs and their occupation of active adsorption sites within the soil, thereby reducing the overall adsorption capacity of the soil [29,51]. (2) MPs may integrate into SOC through the adsorption of organic molecules, thereby enhancing the organically bound fraction of HMs in the soil. These interactions may facilitate the conversion of HMs from bioavailable states into forms that are organically bound [52,53], theoretically reducing the concentration of free HM ions in the soil and decreasing their bioavailability. Conversely, the addition of 10% PE MPs increased the DTPA–Cd and DTPA–Pb content in this study, which contradicts previous findings that indicated the addition of 10% PE MPs decreases the availability of Cd [23]. This may be attributed to the reduction in soil pH caused by 10% PE MPs, which decreased by 1.14 units after 90 days of cultivation compared to the CK (Figure 3A). Research indicates that a decrease in soil pH readily facilitates the conversion of Cd/Pb bound in carbonate complexes and Fe–Mn oxide complexes to ion–exchangeable forms [54,55]. Furthermore, Mantel tests and redundancy analysis provided additional confirmation that pH values exhibit a positive correlation with carbonate binding and Fe–Mn oxide binding (Figure 5).

The Mantel test analysis (Figure 5A,D) revealed a significant positive relationship between SOC content and Cd–F4 and Pb–F4 (*p* < 0.001). These results are consistent with previous research conducted by Yu et al. [53]. Random forest analysis indicated that Cd–F1 and Cd–F3 were significant contributors to DTPA–Cd, while Pb–F1, Pb–F2, and Pb–F3 were major contributors to DTPA–Pb (Figure 5C,F). RDA corroborated these findings (Figure 5B,E). Pb–F1 exhibited a positive correlation with DTPA–Pb, whereas Pb–F2 and Pb–F3 displayed negative correlations with DTPA–Pb. The results of PLS–PM further indicated that changes in PE MP concentrations could not only directly affect bioavailable Cd and Pb by altering soil physicochemical properties but also indirectly influence the forms of Cd and Pb through modifications in soil microbial communities (Figure 7). Prior research has demonstrated that MPs alter the soil physicochemical properties, leading to subsequent changes in microbial communities [56]. Various bacteria and fungi in the soil can utilize MPs or their intermediates as a carbon source for degradation [57]. Additionally, microorganisms can colonize the surface of polyurethane MPs, serving as a habitat [58], which further impacts microbial diversity. Variations in the structure of microbial communities induced by MPs could subsequently affect nutrient cycling and the accessibility of Cd in the soil [22,58]. To further understand the transformation mechanism of PE MPs affecting soil Cd and Pb, we characterized the changes in molecular groups on the surface of soil particles using FTIR, XRD, and XPS. Different doses of PE MPs resulted in varying changes in the molecular groups of the soil. FTIR patterns indicated that 10% concentration of PE MPs significantly affects the Si–O–Si and Fe–O linkages (Figure 6A). According to the XPS spectra, both Fe 2p and Si 2p peak intensities decreased, with the lowest atomic percentages observed under the 10% PE MPs treatment (Figure 6C, Table S2). This suggested that PE MPs influenced the chemical forms of Fe and Si. Previous research has indicated a strong correlation between the presence of iron oxides in soil and the accessibility of HMs. For example, the distances between OH–OH groups in iron oxides closely match the geometry of coordination polyhedra with HMs, thereby contributing to the adsorption of cations and anions at various surface locations [59]. Furthermore, the increase in the number of oxygenated and silica–containing functional groups can provide additional adsorption sites, facilitating the complexation of Pb and Cd [60].

Characteristic diffraction peaks were identified through XRD analysis of soil samples at 2θ = 22.08° and 2θ = 30.96°, corresponding to the precipitation of PbO and PbCO_3_/Cd(OH)_2_. However, the intensity of these diffraction peaks was diminished following a 10% PE MPs treatment. These findings suggest that a high concentration of PE MPs inhibits the oxidation–precipitation processes during Cd^2+^ stabilization while promoting the formation of PbO, PbCO_3_, and Cd(OH)_2_, thereby increasing the solubility and mobility of Cd^2+^ and Pb^2+^. Furthermore, XPS analysis indicated that the C1s spectrum exhibited a shift in the absorption peak for the 10% PE MPs treatment, changing from 288.7 eV to 278.5 eV in the binding energy compared to the CK. This shift implies an interaction between HMs and the oxygen–containing functional groups present on the soil surface [61]. Notably, low doses of PE MPs (0.1% and 1%) reduced the intensities of the O=C–O and C–O peaks compared to the CK, while simultaneously enhancing the intensity of the –OH peaks (Figure 6E). This phenomenon may be attributed to the 0.1% and 1% PE MPs enhancing the adhesion of Cd and Pb to the soil, resulting in a decrease in the intensity and presence of O=C–O and C–O peaks, while concurrently increasing both the height and proportion of the O–H peaks. This suggests that metal cations, which are electron–deficient, diminish the electron cloud density surrounding these functional groups, thereby promoting the fixation of metals [62]. In contrast, a 10% concentration of PE MPs resulted in an increase in the C–O and O=C–O peaks, while the –OH peaks decreased in the high–resolution O1s spectra when compared to lower concentrations of PE MPs. This observation may be attributed to the substantial coverage of active sites related to reactive oxygen by MPs, which significantly diminishes the chemisorption capacity of soil for Cd [29].

## 5. Conclusions

This study revealed that PE MPs influence the bioavailability of soil Cd and Pb through a concentration–dependent effect and elucidated its mechanism. The results indicated that the presence of 0.1% and 1% PE MPs resulted in a reduction in DTPA–extractable Cd and Pb in the soil, while 10% PE MPs increased them. Lower levels of PE MPs (0.1% and 1%) effectively minimized the exchangeable forms of Cd and Pb, thereby facilitating their transformation into more stable compounds, specifically those bound to carbonate, Fe–Mn oxides, and organic matter. Conversely, the higher concentration of 10% PE MPs was found to decrease the fractions of Cd and Pb associated with carbonate and Fe–Mn oxides, while enhancing the exchangeable and residual fractions. The diversity and structure of soil bacterial communities undergo changes under varying PE MP concentrations, with high concentrations exerting a detrimental effect. Correlation analysis revealed strong associations between soil pH, CEC, and SOC content, forms of Cd and Pb, and the bacterial community. FTIR, XRD, and XPS analyses revealed that 0.1% and 1% PE MPs promoted the surface fixation of Cd and Pb with oxygen–containing functional groups, whereas 10% PE MPs inhibited this process and disrupted the formation of PbO, PbCO_3_, and Cd(OH)_2_, thereby increasing the availability of Cd in the soil. Random forest modeling indicated that AP and SOC were the critical factors determining DTPA–Cd and Pb, respectively, and the concentration of PE MPs also significantly affected both DTPA–Cd and Pb. Partial least squares path modeling demonstrated that PE MPs altered the soil physicochemical characteristics and the structure of the bacterial community, ultimately impacting the transformation of Cd and Pb speciation, and these changes were highly dependent on PE MP concentration. This study underscores the intricate and diverse impacts of PE MP pollution on soil ecosystems, and the research elucidates how different concentrations of PE MPs affect the availability of Cd and Pb in the soil environment, and provides further insights into the treatment of Cd and Pb–contaminated soils containing MPs.

## Data Availability

The data of this study are available on request from the corresponding author.

## Supplementary Materials

The following supporting information can be downloaded at: https://www.mdpi.com/article/10.3390/toxics13100901/s1, Supplementary materials Text S1: The detailed procedure for the determination of 5 forms of Cd and Pb; Figure S1: Scanning electron microscope (SEM) images of PE MPs (A), FTIR spectra images of PE MPs (B); Figure S2: Changes in pH in the soil at varying PE MP concentrations. Different letters indicate the significant differences (*p* < 0.05); Figure S3: Changes in AP (A) and AH–N (B) concentrations in the soil over time at varying PE MP concentrations. Different letters indicate the significant differences at the same time point. (*p* < 0.05); Figure S4: Venn diagrams analysis of soil with different PE MP concentration; Figure S5: A: The 10 most important bacterial genera in the soil predicted by random forest models. B: Differences in the relative abundance of the top ten bacterial genera ranked by importance across treatments; Figure S6: The relative abundance of bacterial community in soil at the phylum level; Figure S7: Correlations between soil most important bacterial genera and soil properties. Significant levels are indicated: * *p* < 0.05, ** *p* < 0.01, *** *p* < 0.001; Figure S8: Adsorption of Cd and Pb by PE MPs soil and soil + PE MPs. (The adsorption conditions were set as follows: adsorbent amount = 0.1 g, the concentration of the Cd/Pb solution = 5 mg/L. V = 25 mL, pH 6.8, equilibrium time 1440 min); Table S1: The physicochemical properties of the test soil; Table S2: Atomic percentages (%) for the elements on the sample particle surfaces.
