# Supplementary material for: Concentration-Dependent Effects of Polyethylene Microplastics on Cadmium and Lead Bioavailability in Soil
Source: Toxics. 2025 Oct 21;13(10):901. doi: 10.3390/toxics13100901 (PMC12567860; doi:10.3390/toxics13100901)
Supplement: Supplementary file 1 [file toxics-13-00901-s001.zip › toxics-3842914-supplementary.pdf]

Supplementary Material

Concentration-Dependent Effects of Polyethylene Microplastics on Cadmium and Lead Bioavailability in Soil

Zhenbo Wang[a,b], Sihan Liu[a,b], Peng Zhao [a,b,*], Guangxin Li[a,b], Ran Duan[c], Chang Li[a,b], Haichao Fu[a,b,*]

[a]College of Resources and Environmental Sciences, Henan Agricultural University, Zhengzhou 450002, China

[b]Key Laboratory of Soil Pollution Control and Remediation of Henan Province, Zhengzhou 450002, China

[c]Institute of Quality and Safety for Agro-products, Henan Academy of Agricultural Sciences, Zhengzhou, 450002, China

[*]Corresponding authors: Peng Zhao and Haichao Fu.
E-mail addresses: zhaopeng@henau.edu.cn (P. Zhao), haichaofu@henau.edu.cn (H. Fu).

Supplementary materials Text 1: The detailed procedure for the determination of 5 forms of Cd and Pb.

(1) F1: Exchangeable. After drying and sieving through a 150 μm sieve, 1.0000 g of the soil sample was placed in a 50 mL centrifuge tube, followed by the addition of 8.0 mL of 1.0 M $MgCl_2$ solution (adjusted to pH 7.0 using dilute ammonia and

dilute hydrochloric acid) to the centrifuge tube. The extraction was performed by shaking at room temperature for 2 h, followed by centrifugation at 4000 rpm for 10 min; the supernatant was filtered and the residue was washed with deionised water. The concentration of each HM in the supernatant was measured using AAS.

(2) F2: Carbonate-bound. Eight millilitres of 1.0 M CH_3COONa solution (pH 5.0, adjusted by 1:1 CH_3COOH) was added to the residue obtained after centrifugation in the previous step, extracted for 5 h, and centrifuged at 4000 rpm for 10 min; the supernatant was filtered, washed with deionised water, and recentrifuged. The washing solution was discarded. The concentration of each HM in the supernatant was determined.

(3) F3: Fe/Mn oxide-bound. Twenty millilitres of 0.04 M NH_2OH-HCl solution (dissolved in 25% [V/V] CH_3COOH) was added to the centrifuged residue from the previous step and extracted for 6 h in a water bath at 96 ± 3 °C with intermittent stirring. After centrifugation at 4000 rpm for 10 min, the supernatant was filtered, the residue was washed with deionised water and centrifuged again, and the washing solution was discarded. The concentration of each HM in the supernatant was determined.

(4) F4: Organic matter-bound. A amount of 5 mL of 0.02 M HNO_3 (3.0 mL) and 30% H_2O_2 (HNO_3 adjusted to pH 2.0) were added to the centrifuged residue from the previous step and stirred intermittently for 2 h in a water bath at 85 ± 2 °C. After cooling, 3.2 M CH_3COONH_4 (dissolved in 25% [V/V] HNO_3) (5.0 mL) was

added and agitated for 30 min, followed by centrifugation at 4000 rpm for 10 min. The supernatant was filtered, washed with deionised water, and recentrifuged. The residue was washed with deionised water and recentrifuged, and the washing solution was discarded. The concentration of each HM in the supernatant was measured.

(5) F5: Residual. To the tube containing the centrifuged residue from the previous step, 5.0, 5.0, 1.0, and 5.0 mL of an acid mix (HNO_3, HF, $HClO_4$, and HCl) were added. The heating process was divided into three stages: 100 °C for 60 min, 160 °C for 120 min, and 180 °C for 360 min. The digestion solution was filtered and transferred to volumetric flasks to determine the concentrations of Cd.

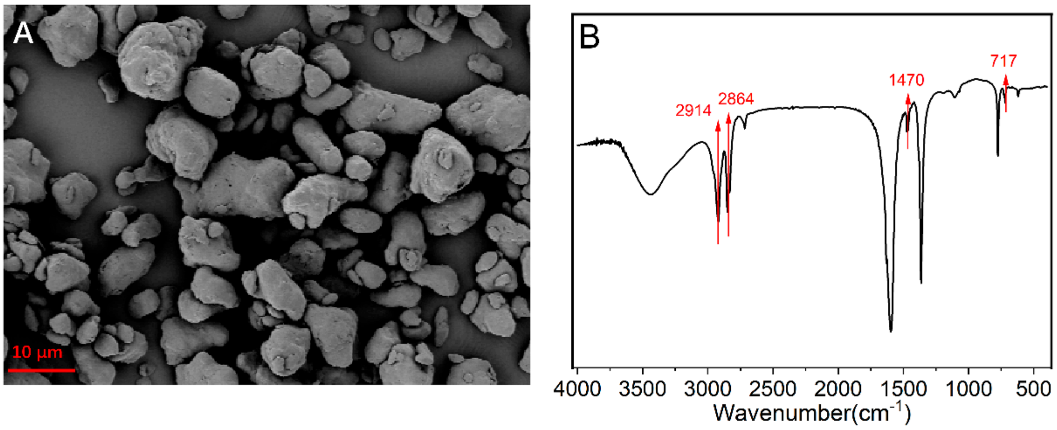

Figure S1. Scanning electron microscope (SEM) images of PE MPs (A) and FTIR spectra images of PE MPs (B).

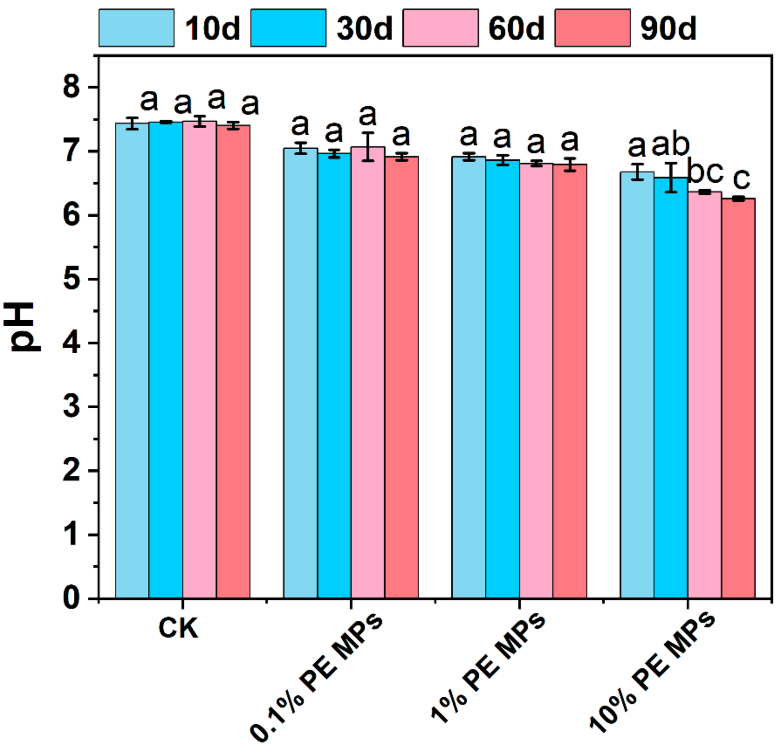

Figure S2. Changes in pH in the soil at varying PE MP concentrations. Different letters indicate the significant differences ($p < 0.05$).

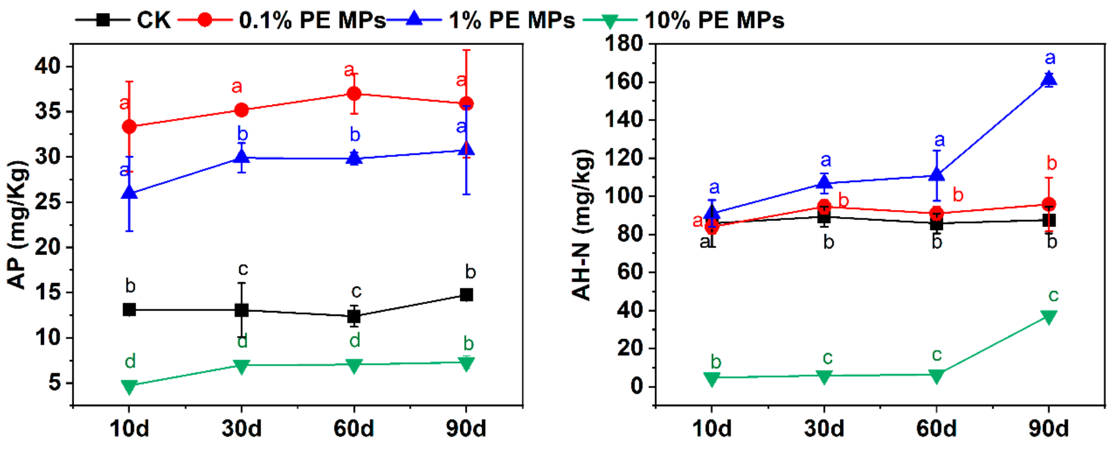

Figure S3. Changes in AP (A) and AH-N (B) concentrations in the soil over time at varying PE MP concentrations. Different letters indicate the significant differences at the same time point ($p < 0.05$).

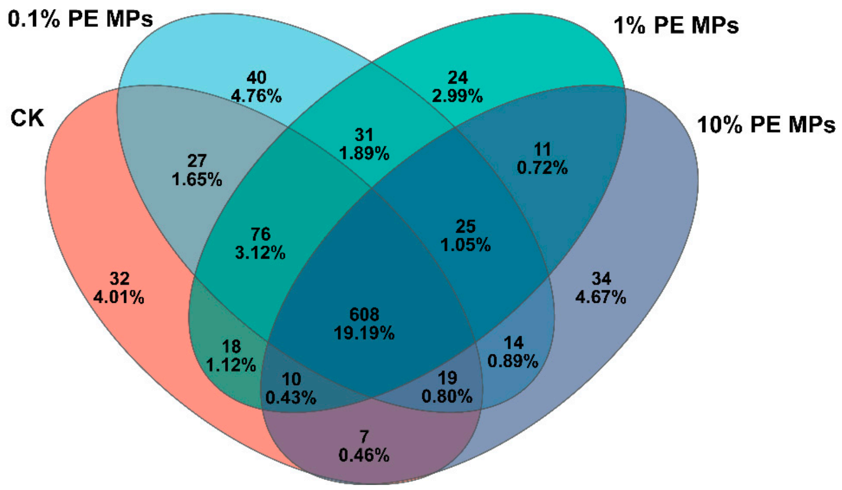

Figure S4. Venn diagram analysis of soil with different PE MP concentrations.

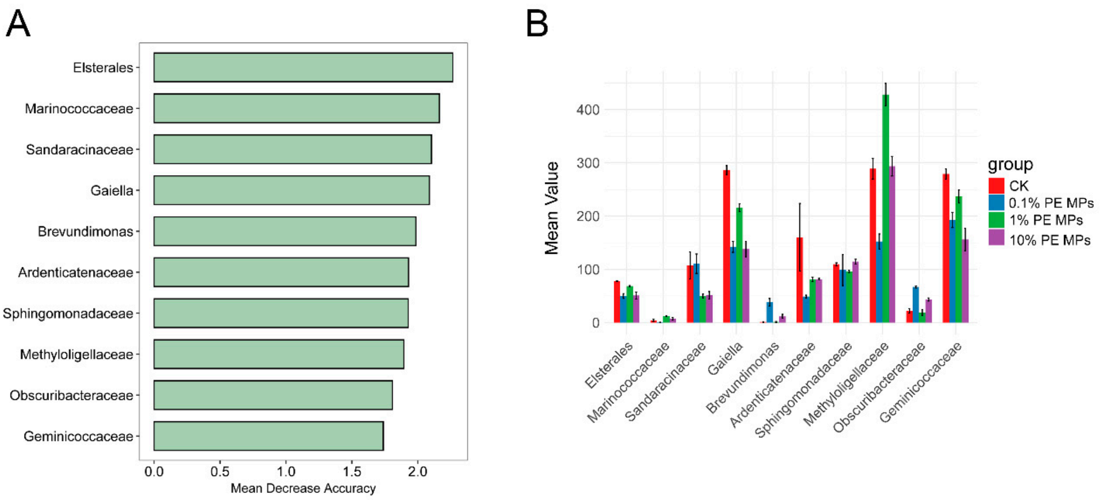

Figure S5. A: The 10 most important bacterial genera in the soil predicted by random forest models. B: Differences in the relative abundance of the top ten bacterial genera ranked by importance across treatments.

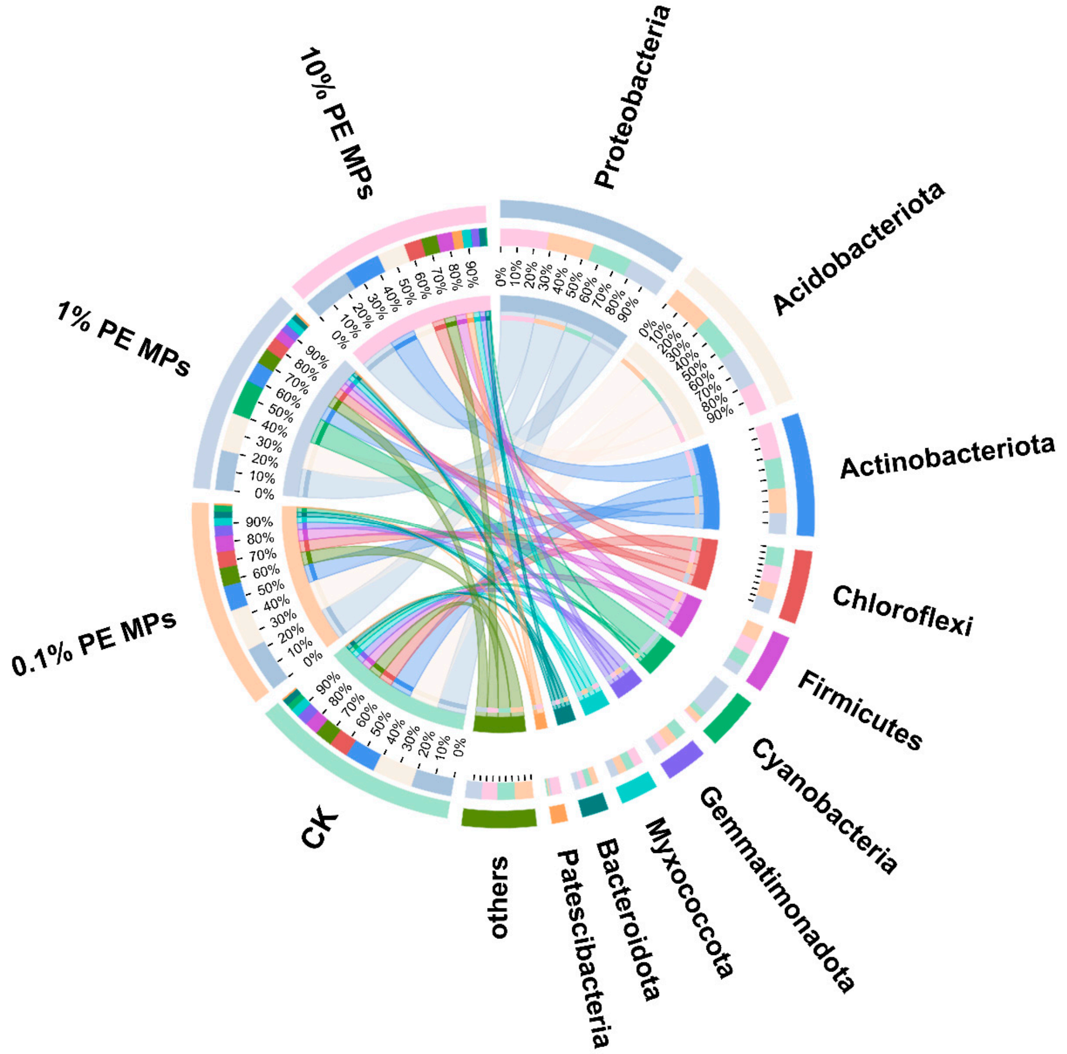

Figure S6. The relative abundance of bacterial community in soil at the phylum level.

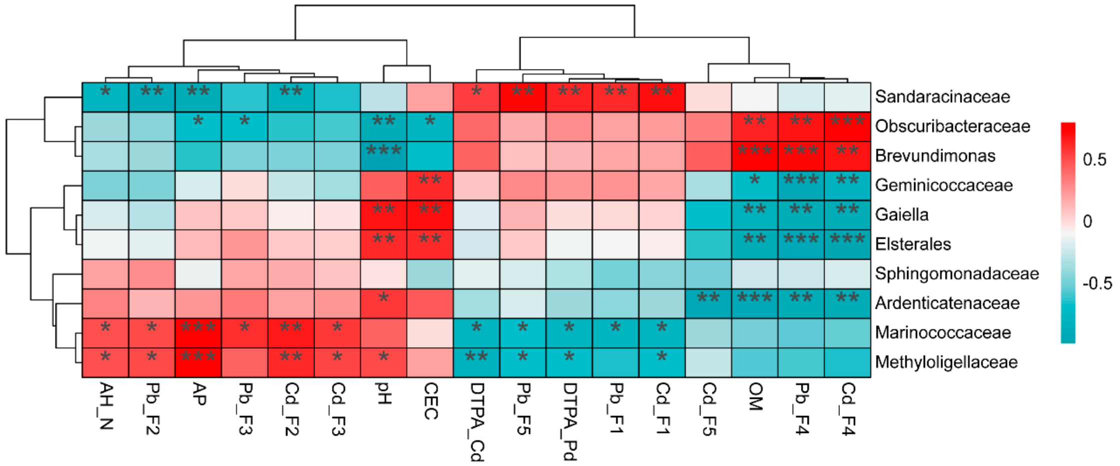

Figure S7. Correlations between soil and most important bacterial genera and soil properties. Significant levels are indicated: *p < 0.05, **p < 0.01, ***p < 0.001.

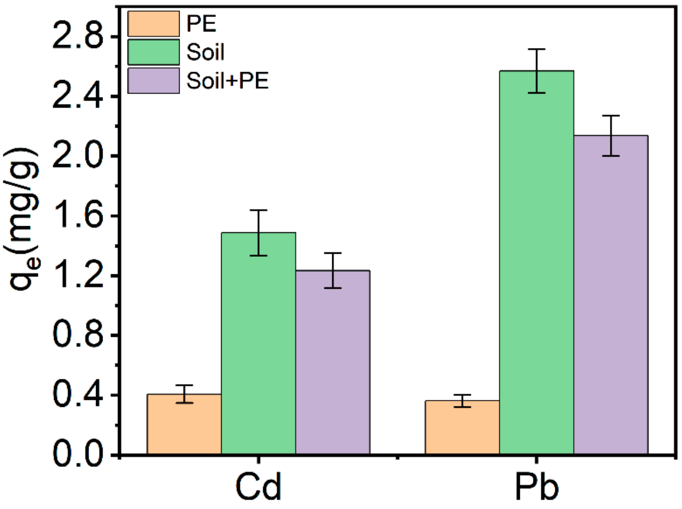

Figure S8. Adsorption of Cd and Pb by PE MPs: soil and soil with 1% PE MPs. Values represent mean ± standard deviation ($n = 3$). (The adsorption conditions were set as follows: adsorbent amount = 0.1 g, the concentration of the Cd/Pb solution = 5 mg/L. V = 25 mL, pH 6.8, equilibrium time 1440 min.)

Table S1. The physicochemical properties of the test soil.

pH	Alkaline nitrogen	Available phosphorus	Available potassium	Organic matter	Cation exchange capacity	Total-Cd	Total - Pb
7.59	65.34 mg/kg	18.03 mg/kg	101.00 mg/kg	13.33 g/kg	12.16 $cmol^+/kg$	5.40 mg/kg	426.00 mg/kg

Table S2. Atomic percentages (%) for the elements on the sample particle surfaces.

Sample	C1s	O1s	Al2p	Si2p	Fe 2p	O/C
CK	6.95	67.24	9.54	14.85	1.43	9.67
0.1% PE MPs	7.16	61.97	10.03	18.76	2.08	8.65
1% PE MPs	11.24	59.4	9.18	18	2.18	5.28
10% PE MPs	45.41	41.5	4.72	8	0.36	0.91
